# Telemedical Education: Training Digital Natives in Telemedicine

**DOI:** 10.2196/jmir.5534

**Published:** 2016-07-12

**Authors:** Akhilesh S Pathipati, Tej D Azad, Kamal Jethwani

**Affiliations:** ^1^ Stanford University School of Medicine Stanford, CA United States; ^2^ Partners HealthCare Connected Health Boston, MA United States; ^3^ Harvard Medical School Boston, MA United States

**Keywords:** telemedicine, medical education, medical school, curriculum reform

## Abstract

Telemedicine plays an important role in the delivery of medical care, and will become increasingly prominent going forward. Current medical students are among the first generation of “digital natives” who are well versed in the incorporation of technology into social interaction. These students are well positioned to apply advances in communications to patient care. Even so, providers require training to effectively leverage these opportunities. Therefore, we recommend introducing telemedicine training into medical school curricula and propose a model for incorporation.

## Telemedicine Overview

Telemedicine refers to the remote delivery of medical care. Doctors have communicated over distance with one another and with patients ever since the advent of the earliest communications tools. However, recent technological advances and a changing health care landscape have transformed telemedicine from a novelty into a booming industry.

Although estimates vary, analysts project the telemedicine market to be US $20-$30 billion by 2020, with more than 100 million e-visits happening annually [1]. Nearly half of all hospitals in the United States have active telemedicine programs and are employing increasingly sophisticated tools [2]. Traditional models focus on telephone, email, and videoconferencing to care for minor conditions. These modalities remain relevant, but the field has rapidly added capabilities and indications [3]. Telemedicine includes diagnostics, treatment, monitoring, consultation, and education among other domains.

Telemedicine has become a fundamental piece of American health care delivery because it helps address issues of both health care costs and access. Moving forward, digital health capabilities will only continue to grow. In order to most effectively leverage these tools, we must ensure providers use them effectively and appropriately. Today’s medical trainees are well versed in technology, but the practice of telemedicine is not necessarily intuitive. Therefore, we advocate the introduction of telemedicine training into medical schools.

## Ensuring High-Quality Care

Few among us would claim the ability to conduct an engaging conversation as a guarantee of prowess in eliciting a comprehensive patient history. Similarly, we should refrain from assuming that digital native physicians will deliver high-quality telemedical care without formal and systematic training. Current research suggests that telemedicine has a great deal of promise, but successful studies are typically carried out in academic medical centers by a limited number of well-trained doctors [4]. Other studies have shown that telemedicine can lead to mixed-quality care. For instance, Mehrotra et al [5] found that e-visits had roughly the same treatment outcomes as in-person visits for sinusitis and urinary tract infections, but e-visits had higher rates of antibiotic prescription. Schoenfeld et al [6] found considerable variation in the quality of care provided by commercial telemedicine companies. As more patients are seen remotely and indications for telemedicine become more complex, we need to train physicians to offer digital care on par with in-person consultation.

Medical education must recognize the intrinsic differences between the practice of traditional medicine and that of telemedicine. For instance, it is difficult to remotely carry out a physical exam, which fundamentally changes the diagnostic process. Technological limitations may cause marked variation in data quality between clinic and remote visits. A patient’s self-reported blood pressure from home may differ from that measured by a nurse in clinic. Providers need to be able to judge those differences. Telemedicine has its limitations in other dimensions as well. Pain management is difficult to gauge from afar. Complex diagnoses and the initial phases of patient education may be better done in person. The nature of the doctor-patient relationship is different. The list goes on.

Given these limitations, practitioners must be able to determine when telemedicine is appropriate and how to optimally process information when they see patients remotely. They must also understand how to navigate the many medicolegal issues that remain in telemedicine, including the role of Health Insurance Portability and Accountability Act regulations, restrictions due to licensing laws, and issues regarding malpractice. Telemedicine is a rapidly evolving field with many stakeholders and murky regulation; providers must learn how to interact with such a system.

## The Role of Telemedical Training

Formal training is the best way to teach providers how to approach the challenges and opportunities inherent in telemedicine. We propose that this training should begin in medical school.

Today’s medical trainees are the first generation of digital natives—individuals who grew up surrounded by digital technology and are therefore comfortable processing information in an electronic world. This fact is not enough to guarantee high-quality telemedical care. Formal training can extend and amplify the impact that telemedicine brings to health care. Consider the analogy of a young athlete: the first time a tennis racket is in her hand, it is an extension of her right arm, her forehand develops easily, and she demonstrates the footwork and court instincts of a player twice her age. She’s a natural. However, the distance between her innate ability and a professional career, let alone a legacy of greatness akin to that of Serena Williams, is vast. Dedicated training and repeated practice will determine whether she competes at the game’s highest level. Current medical students’ inherent comfort with technology should be nurtured through structured training. Without this, providers will be ill prepared to take advantage of new innovations in telemedicine.

With this in mind, we propose incorporating telemedical training into the standardized medical school curriculum. We have an opportunity to translate students’ familiarity with technology into superior medical care. Creating a formal training program will allow students to directly compare and contrast telemedicine with traditional medicine, recognize when to use it, and learn best practices. Placing the training program in medical schools would ensure that all new doctors have that ability. To ensure high-quality telemedical care, we must train students to practice telemedicine with the same level of skill they demonstrate delivering traditional care.

## A Model for Incorporation

Although creating any new medical education program can seem daunting, we believe telemedicine education can be readily incorporated. Nascent efforts that expose medical trainees to telemedicine have already proven to be successful. For instance, dermatology residents and medical students on a dermatology rotation at the Denver Department of Veterans Affairs Medical Center participated in teledermatology consultations with faculty oversight [7]. Trainees reported that it was a valuable educational tool, both in terms of developing medical knowledge as well as improving their ability to provide patient care. Pilot programs at other institutions have also begun to evaluate the role of telemedicine in medical education [8-9].

To date, telemedicine training has been limited to small research settings, such as those described previously. We believe it should become a more prominent part of the medical school curriculum moving forward. Two of the authors (ASP and TDA) are students at the Stanford University School of Medicine. As such, we will use Stanford’s curriculum as a theoretical model for how telemedicine education can be built into medical training.

The first two years at Stanford are the “preclinical” years, during which students take classes in the basic sciences, as well as a clinical skills class known as “Practice of Medicine” (POM). POM takes place during two 4-hour sessions each week (8 hours/week total) throughout the first two years. Students attend lectures on the process of clinical reasoning, learn how to do a history and physical exam with standardized patients (ie, actors who are pretending to be patients), work through patient cases in groups, and spend several afternoons in Stanford Hospital honing those skills with real patients. The third and fourth years of medical school are the “clinical” years, during which students rotate through various different specialties and participate in patient care.

Telemedicine training may be incorporated into both phases of medical school. During the preclinical years, one POM session every 2 months could be modified such that students must interact with patients electronically rather than in person. The clinical reasoning lectures that take place before these sessions should highlight the salient differences between electronic and traditional encounters, such as how to conduct an encounter without the physical exam, overviews of available health technologies, etc. Further research should be conducted on how to conduct a safe and effective virtual exam [10], which can then be translated into best practices.

The process of setting up a telemedicine experience during rotations is even more intuitive. Many specialties are amenable to telemedicine, including radiology, dermatology, and primary care, among others. Students rotating in these specialties should be required to complete 10 to 20 hours on “digital call,” during which they would participate in electronic encounters with faculty supervision, learn about remote monitoring tools, and develop the background necessary to be an effective provider in the future. Schools may also consider the idea of a “digital health rotation,” in which students would spend 2 to 4 weeks learning how new tools can be applied in practice across fields. Granted, not all medical schools currently have the technological infrastructure in place to offer a digital call experience, but we expect those capabilities to develop as telemedicine continues to grow.

Although these suggestions are based on Stanford’s curriculum, nearly every medical school in the country has a clinical skills class during the preclinical years, and clinical rotations during the final two years of medical school. Therefore, we expect the model to be generalizable to most medical schools in the United States. Further research should be conducted on specific skills and techniques that go into a safe and effective virtual encounter.

American health care is in the midst of a transformation, and telemedicine will be a cornerstone of the result. Proper training will allow us to maximize its potential.

## Abbreviations

POM: Practice of Medicine
